# Targeting SPHK1/PBX1 Axis Induced Cell Cycle Arrest in Non-Small Cell Lung Cancer

**DOI:** 10.3390/ijms232112741

**Published:** 2022-10-22

**Authors:** Zhoujun Lin, Yin Li, Xiao Han, Zhenkun Fu, Zhenhuan Tian, Chenggang Li

**Affiliations:** 1 State Key Laboratory of Medicinal Chemical Biology and College of Pharmacy, Nankai University, No. 38 Tongyan Road, Jinnan District, Tianjin 300350, China; 2 Heilongjiang Provincial Key Laboratory for Infection and Immunity, Department of Immunology, Wu Lien-Teh Institute, Heilongjiang Academy of Medical Science, Harbin Medical University, Harbin 150081, China; 3 Department of Thoracic Surgery, Peking Union Medical College Hospital, No. 1 Shuaifuyuan, Dongcheng District, Beijing 100730, China

**Keywords:** non-small cell lung cancer, SPHK1, PBX1, S1PR3, cell cycle

## Abstract

Non-small cell lung cancer (NSCLC) accounts for 85~90% of lung cancer cases, with a poor prognosis and a low 5-year survival rate. Sphingosine kinase-1 (SPHK1), a key enzyme in regulating sphingolipid metabolism, has been reported to be involved in the development of NSCLC, although the underlying mechanism remains unclear. In the present study, we demonstrated the abnormal signature of SPHK1 in NSCLC lesions and cell lines of lung cancers with a potential tumorigenic role in cell cycle regulation. Functionally, ectopic Pre-B cell leukemia homeobox-1 (*PBX1*) was capable of restoring the arrested G1 phase induced by *SPHK1* knockdown. However, exogenous sphingosine-1-phosphate (S1P) supply had little impact on the cell cycle arrest by *PBX1* silence. Furthermore, S1P receptor S1PR3 was revealed as a specific switch to transport the extracellular S1P signal into cells, and subsequently activated PBX1 to regulate cell cycle progression. In addition, Akt signaling partially participated in the SPHK1/S1PR3/PBX1 axis to regulate the cell cycle, and the Akt inhibitor significantly decreased PBX1 expression and induced G1 arrest. Targeting SPHK1 with PF-543 significantly inhibited the cell cycle and tumor growth in preclinical xenograft tumor models of NSCLC. Taken together, our findings exhibit the vital role of the SPHK1/S1PR3/PBX1 axis in regulating the cell cycle of NSCLC, and targeting SPHK1 may develop a therapeutic effect in tumor treatment.

## 1. Introduction

Lung cancer is one of the most common malignant tumors and a leading cause of cancer-related morbidity and mortality worldwide, with nearly 2 million new cases each year and an extremely low 5-year survival rate [1,2]. Non-small cell lung cancer (NSCLC), a major type of lung cancer accounting for 85~90% of the cases, is generally divided into adenocarcinoma, squamous cell carcinoma and large cell carcinoma [3]. Currently, the most effective treatment for NSCLC includes surgery combined with radiotherapy and chemotherapy, although it comes with a dismal prognosis [4]. Hence, exploring related genes and finding important pathways in the development of NSCLC will be critical to understand the malignancy of tumors and to improve the survival of NSCLC patients.

Biologically active lipids have emerged as signaling molecules with pleiotropic effects on important cellular processes, including cell proliferation, migration and pluripotency [5]. Sphingosine kinase-1 (SPHK1) is a primary member of the SPHK family, catalyzing the production of sphingosine-1-phosphate (S1P) [6]. The S1P-related pathway orchestrates numerous cellular processes essential for cell proliferation and survival through five G-protein-coupled receptors (GPCRs), S1PR1-5, in an inside-out manner or as an intracellular signaling lipid messenger [7]. The accumulated data have indicated that SPHK1 is an integral component of the cancer cell network and can be “hijacked” for cell renewal and survival, including in breast, ovarian and lung cancer [8,9,10]. Masayuki et al. demonstrated a critical role for circulating S1P produced by tumors and the SPHK1/S1P/S1PR1 axis in obesity-related inflammation, the formation of lung metastatic niches and breast cancer metastasis [11]. In bladder cancer, elevated SPHK1 expression was also proved to enhance the chemoresistance to cisplatin and contribute to poor survival rates in patients [12]. In addition, previous studies have already linked the high expression of SPHK1 with tumor progression and the poor survival of patients with NSCLC [13], but the molecular mechanism still needs further investigation.

Pre-B cell leukemia homeobox-1 (PBX1) is a member of the TALE family of atypical homeodomain transcription factors, initially identified as part of the chimeric transcription factor resulting from the chromosomal translocation t (1;19) in pre-B cell acute lymphoblastic leukemias [14,15]. PBX1 or the products of the fusion between *E2A* and *PBX1* genes involved in regulatory networks are frequently equipped tumors with self-renewal, repopulation and resistance to chemotherapeutics [16,17]. Luca et al. identified PBX1 amplification as a functional hallmark of aggressive ERα-positive breast cancers [18]. In ovarian cancer and myeloproliferative neoplasm, PBX1 has been shown to participate in maintaining cancer stem cell-like phenotypes and promoting platinum resistance, at least partially, through its intricate interaction with the JAK2/STAT3 signaling network [16,19]. Here, we explored the role of PBX1 in SPHK1-promoted cell cycle progression in NSCLC. We uncovered that the SPHK1/S1PR3/PBX1 axis and a feedback interaction loop between PBX1 and S1PR3, were potential factors for the proliferation and development of NSCLC, which might bring new insights into NSCLC treatment.

## 2. Results

### 2.1. Accumulation of SPHK1 in NSCLC

**Figure 1:**
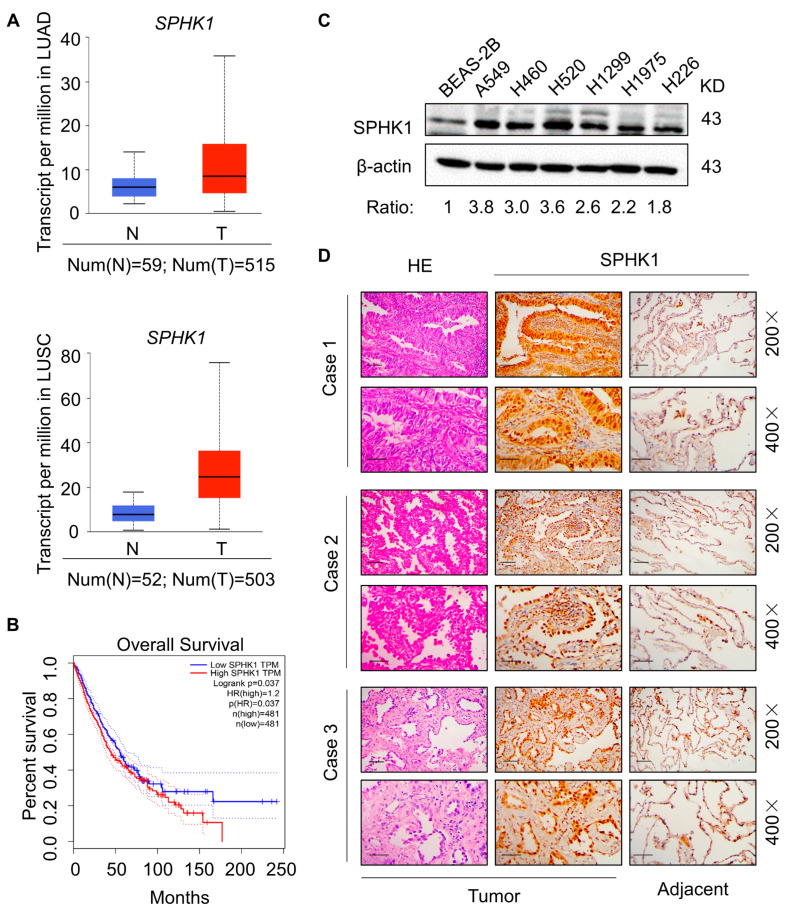
Accumulation of SPHK1 in NSCLC. (**A**) Comparison of *SPHK1* mRNA expression in tumor lesions and normal (healthy) tissues from TCGA lung adenocarcinoma (LUAD) dataset and lung squamous cell carcinoma (LUSC) dataset. (**B**) Kaplan–Meier analysis of estimated overall survival of subjects with lung cancers with high and low SPHK1 expression in TCGA dataset (*n* = 962; *p* < 0.05). (**C**) Western blot of SPHK1 protein in cultured NSCLC cell lines (A549, H460, H520, H1299, H1975, H226), and in normal epithelial cells (BEAS-2B), β-actin was used as a loading control. (**D**) H&E staining and immunohistochemical staining of SPHK1 in NSCLC tumor tissues and paired adjacent normal tissues. Representative image, magnification 200×, bar = 100 µm, 400×, bar = 50 µm.

First, the Cancer Genome Atlas database (TCGA) validated that both in lung adenocarcinoma and squamous cell carcinoma (due to the lack of large cell carcinoma sample sizes, there is no corresponding database), the expression of *SPHK1* in tumor tissues was notably up-regulated (Figure 1A). Kaplan–Meier survival analysis showed that high SPHK1 expression predicted poorer overall survival (OS) than that with weak SPHK1 expression (Figure 1B). Based on the database analysis, we then examined endogenous SPHK1 expression in various NSCLC cell lines, including A549, H460, H520, H1299, H1975 and H226. Western blot showed that all expressed relatively high levels of SPHK1 in comparison with normal lung alveolar epithelial cell line BEAS-2B (Figure 1C). Further evaluation of SPHK1 levels in paraffin-embedded, archived clinical tumor specimens of NSCLC cases, using IHC analysis with an antibody against human SPHK1, revealed significantly elevated SPHK1 levels in NSCLC cases compared to the matched adjacent tissue (Figure 1D). Collectively, these data demonstrated that SPHK1 accumulated in NSCLC and might serve as a potential prognostic marker for patients with NSCLC.

### 2.2. SPHK1 Deletion Inhibited Cell Proliferation in NSCLC

**Figure 2:**
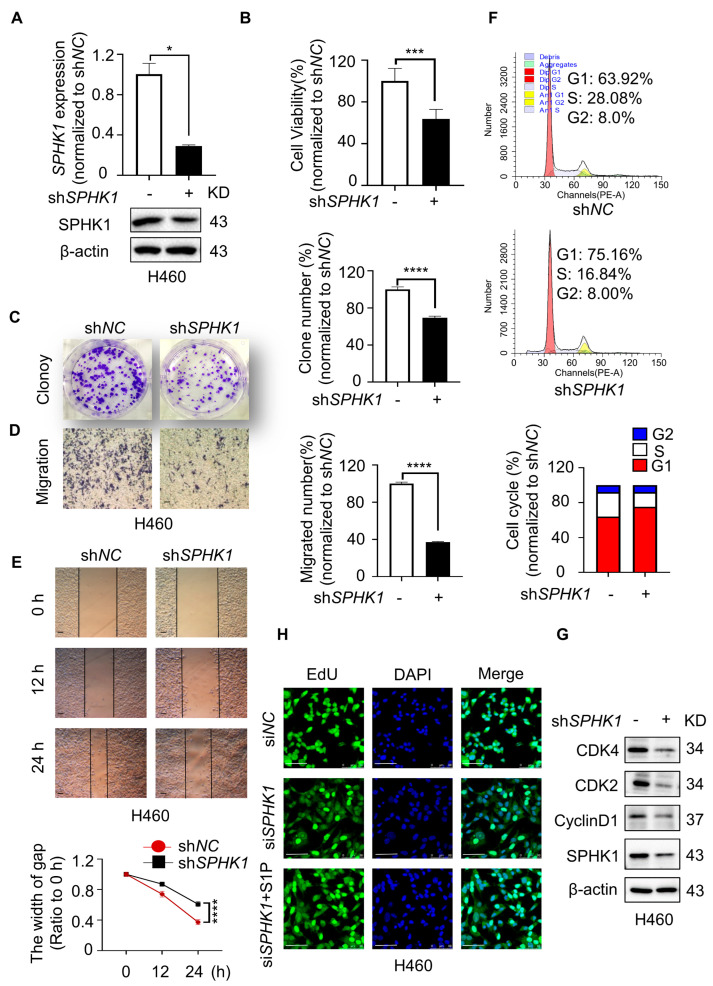
*SPHK1* deletion inhibited cell proliferation in NSCLC. (**A**) *SPHK1* knockdown efficiency was validated on the gene and protein expression in H460 cells by RT-qPCR and Western blotting, respectively. * *p* < 0.05, sh*NC* versus sh*SPHK1*, Student’s *t*-test. (**B**) Cell viability of *SPHK1* knockdown of H460 cells was measured by MTT assay. *** *p* < 0.001, sh*NC* versus sh*SPHK1*, Student’s *t*-test. (**C**) The effect of *SPHK1* knockdown on the colony formation of H460 cells was investigated by colony formation assay. Representative image, magnification 4×. **** *p* < 0.0001, sh*NC* versus sh*SPHK1*, Student’s *t*-test. (**D**) The effect of *SPHK1* knockdown on cell migration of H460 cells was investigated by transwell assay. Representative image, magnification 40×, bar = 200 µm. **** *p* < 0.0001, sh*NC* versus sh*SPHK1*, Student’s *t*-test. (**E**) Wound-healing assay was performed in H460 cells with different *SPHK1* expression levels. Representative image, magnification 40×, bar = 200 µm. **** *p* < 0.0001, sh*NC* versus sh*SPHK1* at 24 h, Student’s *t*-test. (**F**) Flow cytometry analyzed cell cycle distribution in H460 cells with *SPHK1* knockdown. (**G**) Western blot analyzed the changes in SPHK1, CDK4, CDK2 and CyclinD1 in H460 sh*NC* and H460 sh*SPHK1* cells. (**H**) The effect of *SPHK1* silence and exogenous S1P (5 μM) treatment on cell proliferation examined by EdU assay in H460 cells. DAPI was used for nuclear staining, magnification 630×, bar = 50 µm.

Given the significant expression difference and clinical relevance of SPHK1, we further evaluated the direct roles of SPHK1 in NSCLC cells. Then, *SPHK1*-targeting shRNA (sh*SPHK1*) or corresponding controls (sh*NC*) were used to establish a stable *SPHK1*-knockdown cell line in H460 cells. The knockdown efficiency was evaluated by RT-qPCR and Western blot analysis (Figure 2A). Results showed that *SPHK1* deletion significantly suppressed cell viability, colony formation and migration in H460 cells, as confirmed by MTT, colony formation, transwell and wound-healing assays (Figure 2B–E). The cell cycle is an indispensable progression for cell proliferation, and central to this process are cyclin-dependent kinases complexed with the cyclin proteins [20,21]. It was revealed that H460 cells with *SPHK1* silence induced G1/S phase arrest, consistent with the significantly decreased expression of G1 phase cyclin markers CDK4, CDK2 and CyclinD1 (Figure 2F,G). SPHK1 catalyzes the phosphorylation of sphingosine to form S1P, a novel lipid messenger with both intracellular and extracellular functions to promote cell proliferation and survival [22,23]. To intuitively evaluate the effect of S1P, we then detected the proliferative ability of H460 cells with *SPHK1* silence in the presence or absence of exogenous S1P. EdU assays showed that the inhibitory effect on cell proliferation induced by *SPHK1* deletion was notably reversed by the exogenous S1P (Figure 2H). Thus, these findings strongly supported the oncogenic role of SPHK1 in promoting a proliferative phenotype in NSCLC cells.

### 2.3. Pharmacological Inhibition of SPHK1 by PF-543 Induced Cell Cycle Arrest

**Figure 3:**
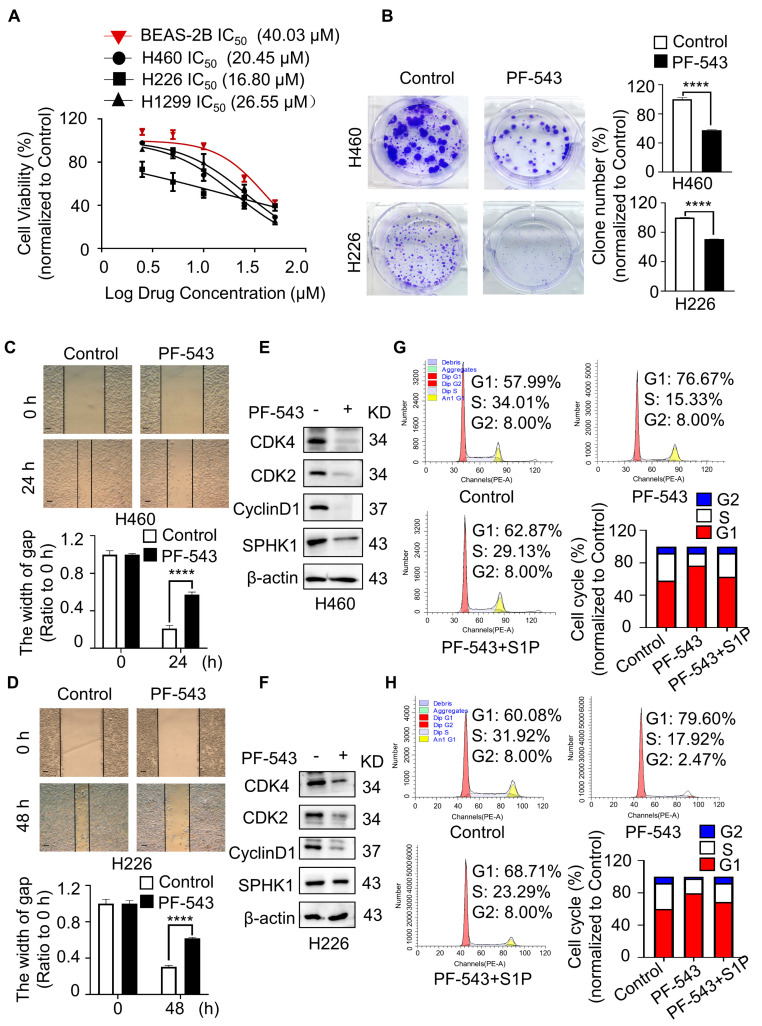
Pharmacological inhibition of SPHK1 by PF-543 induced cell cycle arrest. (**A**) The viability of H460, H226 and H1299 cells and normal lung epithelial BEAS-2B cells was determined by MTT assay after the cells treated with PF-543 at the indicated concentrations for 48 h. The IC_50_ of PF-543 for each cell line was calculated according to the cell viability value. (**B**) Colony formation capacity was investigated in H460 and H226 cells with 15 μM PF-543 treatment. Representative image, magnification 4×. **** *p* < 0.0001, Control versus PF-543, Student’s *t*-test. (**C**,**D**) Wound-healing assays performed in H460 and H226 cells exposed to 15 μM PF-543 treatment. Representative image, magnification 40×, bar = 200 µm. **** *p* < 0.0001, Control versus PF-543 at 24 h or 48 h, Student’s *t*-test. (**E**,**F**) Western blot analyzed the changes in SPHK1, CDK4, CDK2 and CyclinD1 in H460 and H226 cells exposed to 15 μM PF-543 treatment. (**G**,**H**) Flow cytometry analyzed cell cycle distribution in H460 cells and H226 cells. Cells were exposed to 15 μM PF-543 or 5 μM S1P treatment for 48 h.

PF-543, a novel and selective inhibitor of SPHK1 among all the SK inhibitors, exerted potent antiproliferation and cytotoxic effects applied in multiple cancer treatments [24]. A study demonstrated that PF-543 alleviated lung injury caused by sepsis in acute ethanol intoxication rats by suppressing the SPHK1/S1P/S1PR1 signaling pathway [25]. To further characterize the oncogenic role of SPHK1 in NSCLC cells, MTT assay was performed to test the effect of PF-543 on cell survival. Results showed that compared with BEAS-2B, treatment with PF-543 led to substantial declines in cell viability, represented by H460, H226 and H1299 cells, suggesting that cancer cells were more sensitive to SPHK1-inhibition than normal lung epithelial cells, with a half maximal inhibitory concentration (IC_50_) of 20.45 µM in H460 cells, 16.80 µM in H226 cells and 26.55 µM in H1299 cells, respectively (Figure 3A). Based on the MTT assay, we then chose a 15 μM concentration of PF-543 for the following cell experiments, which was lower than the IC_50_ value. Likewise, drug-treated H460 and H226 cells displayed lower clone formation and migration abilities, as confirmed by clone formation and wound-healing assays (Figure 3B–D). Moreover, protein and flow cytometry analysis revealed PF-543 treatment significantly induced G1/S phase arrest in H460 and H226 cells, while exogenous S1P replenishment promoted the cell cycle transition from the G1 phase to the S phase, as revealed by a decreased percentage of G1 phase cells and an increased percentage of S phase cells (Figure 3E–H). To sum up, these pharmacological experiments established the oncogenic role of SPHK1 in promoting proliferation and the cell cycle in NSCLC.

### 2.4. PBX1-Mediated SPHK1 Inhibition Induced G1/S Stage Cell Cycle Arrest

**Figure 4:**
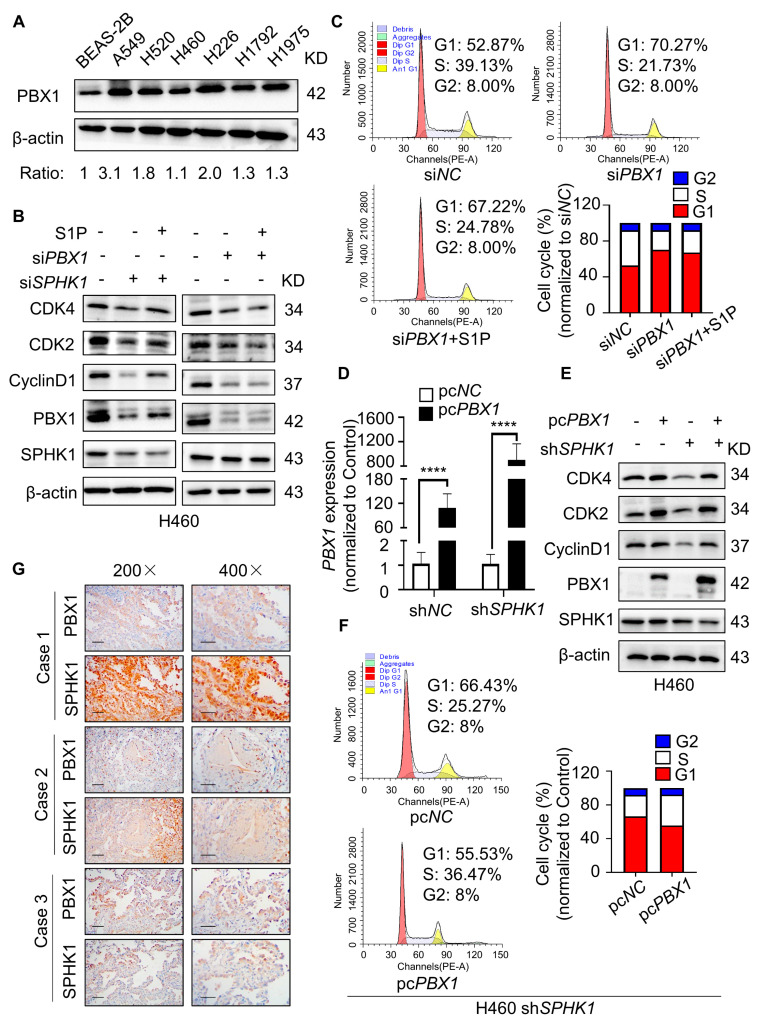
PBX1-mediated SPHK1 inhibition induced G1/S stage cell cycle arrest. (**A**) Western blot of PBX1 protein in cultured NSCLC cell lines (A549, H520, H1299, H460, H226, H1792 and H1975) and in normal epithelial cells (BEAS-2B), β-actin was used as a loading control. (**B**) Western blot detected the change in SPHK1, PBX1, CDK4, CDK2 and CyclinD1 in H460 cells with *SPHK1* or *PBX1* silence (siRNA 50 nM) in the presence or the absence of S1P (5 μM). (**C**) Flow cytometry analyzed cell cycle distribution in H460 cells with *PBX1* silence (siRNA 50 nM) in the presence or the absence of S1P (5 μM). (**D**) *PBX1*-overexpressing efficiency was validated on *PBX1* gene in H460 sh*NC* and H460 sh*SPHK1* cells by RT-qPCR. **** *p* < 0.0001, pc*NC* versus pc*PBX1* in sh*NC* group and pc*NC* versus pc*PBX1* in sh*SPHK1* group, Student’s *t*-test. (**E**) Western blot detected proteins of SPHK1, PBX1, CDK4, CDK2 and CyclinD1 in H460 sh*NC* and H460 sh*SPHK1* cells with different *PBX1* expression. (**F**) Flow cytometry analyzed cell cycle distribution in H460 sh*SPHK1* cells with different *PBX1* expression levels. (**G**) Identification of the correlation between protein levels of SPHK1 and PBX1 in NSCLC tissues with IHC analysis, magnification 200×, bar = 100 µm, 400×, bar = 50 µm.

The foregoing data have clearly demonstrated that the regulation of SPHK1, regardless of *SPHK1* depletion or pharmacological inhibition, resulted in G1/S phase arrest in NSCLC cells, which made us curious about how SPHK1 participates in the cell cycle regulation. Based on the sphingolipid metabolism and its downstream effectors, we focused on the transcription factor PBX1. It has been suggested that PBX1 overexpression promotes cell proliferation, cell cycle progression and osteogenesis [26]. Meanwhile, the transcriptional activation of *E2F5*, a cell cycle regulator, was also coregulated by E2A-PBX1, RUNX1 and MED1 [27]. In addition, a study revealed that another cell cycle regulator CyclinD1 was subjected to the regulation of transcription factor PBX1 in clear cell renal carcinoma [28]. Our experimental results showed the relatively high endogenous PBX1 expression in various NSCLC cell lines compared to BEAS-2B (Figure 4A), but we wondered whether there was a relationship between SPHK1 and PBX1 in the cell cycle regulation. To prove the association, H460 cells were transiently transfected with si*SPHK1* or si*PBX1* compared to their negative control si*NC*, respectively. Western blot analysis showed that *SPHK1* silence significantly down-regulated the expression of PBX1, while *PBX1* suppression made no reference to SPHK1 expression (Figure 4B). Regarding the cell cycle process, protein and flow cytometry analysis further revealed that the addition of exogenous S1P moderately up-regulated the PBX1 expression and G1 phase-related markers in cells with *SPHK1* silence; whereas S1P replenishment did not contribute to the cell cycle arrest and the activation of cyclin markers in cells with *PBX1* silence, which was quite different from S1P promoting the cell cycle in *SPHK1* depletion cells (Figure 4B,C). These results made us speculate as to whether SPHK1 was in the upstream of PBX1 and might regulate the cell cycle through PBX1. Then we used *SPHK1*-knockdown (sh*SPHK1*) and the corresponding control (sh*NC*) H460 cells to generate cells with different levels of *PBX1* overexpression (pc*PBX1*) or its negative control (pc*NC*). RT-qPCR showed the transfection efficiency in sh*SPHK1* cells was approximately nine-fold that of sh*NC* cells (Figure 4D). Protein and flow cytometry analysis supported that ectopic *PBX1* expression was capable enough to rescue the proliferative potential of cells with *SPHK1* suppression, as confirmed by the decreased proportion of G1 phase and increased S phase in sh*SPHK1* cells with *PBX1*-overexpressing compared with the control group (Figure 4E,F). The similar role of PBX1 mediated in SPHK1-regulated cell cycle progression was also corroborated in another NSCLC cell line H226 (Figure S1A,B,G). In addition, we performed IHC staining with both anti-SPHK1 and anti-PBX1 antibodies on NSCLC tumor specimens to measure the correlation between the two molecules. Cases showed high expression of SPHK1 accompanied with higher PBX1 expression at the same location of pathological sections, suggesting a potentially positive correlation between SPHK1 and PBX1 on protein level (Figure 4G). Hence, these results supported that PBX1 may be downstream of SPHK1 and indispensable for the SPHK1-regulated cell cycle process in NSCLC cells.

### 2.5. S1PR3/PBX1 Axis Regulated Cell Cycle Progress

**Figure 5:**
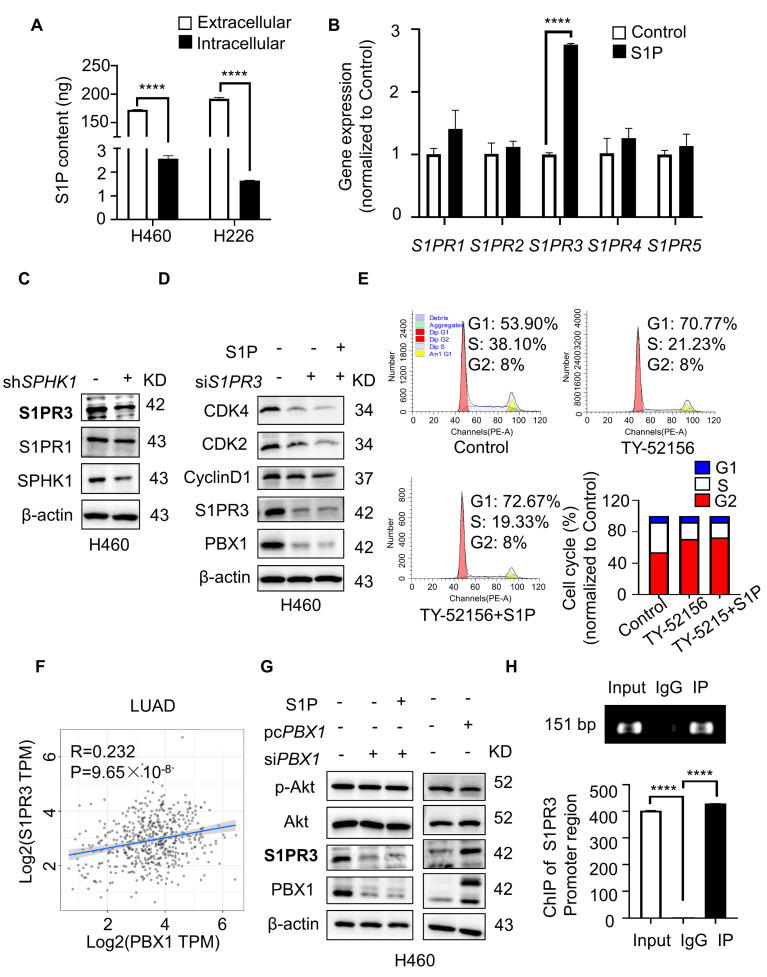
S1PR3/PBX1 axis regulated cell cycle progress. (**A**) Extracellular and intracellular S1P contents were detected by S1P ELISA kit. **** *p* < 0.0001, extracellular versus intracellular, Student’s *t*-test. (**B**) RT-qPCR detected possible *S1PRs* expression in H460 cells after stimulation with exogenous S1P (5 μM) for 24 h. **** *p* < 0.0001, control versus S1P, Student’s *t*-test. (**C**) Western blot detected the expression of S1PR3, S1PR1 and SPHK1 in H460 sh*NC* and sh*SPHK1* cells. (**D**) Western blot detected the expression of S1PR3, SPHK1, PBX1, CDK4, CDK2 and CyclinD1 in H460 cells with *S1PR3* silence (siRNA 50 nM) in the presence or the absence of S1P (5 μM). (**E**) Flow cytometry analyzed cell cycle distribution in H460 cells with TY-52156 (5 μM) treatment in the presence or the absence of S1P (5 μM). (**F**) Gene correlation analysis between *S1PR3* and *PBX1* of LUAD in TIMER database. (**G**) Western blot detected the expression of p-Akt, Akt, S1PR3 and PBX1 in H460 cells with *PBX1* silence or *PBX1* overexpression. (**H**) RT-PCR was performed to determine gene abundance of S1PR3 promoter region in the different groups, which were immunoprecipitated using an anti-PBX1 antibody in H460 cells. **** *p* < 0.0001, Input versus IgG and IP versus IgG, Student’s *t*-test.

In our study, we discovered that H460 and H226 cells contained richer extracellular S1P levels compared with intracellular levels (Figure 5A), and replenishing exogenous S1P reversed the effect of cell cycle arrest induced by SPHK1 suppression, leading us to speculate that S1P produced by SPHK1 might act via an “inside-out” manner. In tumor cells, S1PR1 was identified as a potential target to block STAT3 signaling in activated B cell-like diffuse large B-cell lymphoma [29]. S1PR2 signaling induced AML growth and was shown to activate ezrin–radixin–moesin (ERM) proteins to induce motility and the invasion of HeLa cells in culture [30]. S1PR3 signaling promoted aerobic glycolysis by the YAP/c-MYC/PGAM1 axis in osteosarcoma [31]. Therefore, we then investigated the possible role of S1PRs in the SPHK1-regulated cell proliferation of NSCLC. After exposing cells to exogenous S1P for 24 h, we detected the gene transcription of *S1PR_1-5_* by RT-qPCR, and results showed that exogenous S1P stimulation prominently up-regulated the transcription level of *S1PR3* compared to other receptors (Figure 5B). Consistent with gene results, H460 cells with *SPHK1* silence had significantly lower S1PR3 expression compared to that of S1PR1 (Figure 5C). To further characterize the effect of S1PR3 in the SPHK1/PBX1 axis of NSCLC, H460 cells were transiently transfected with siRNAs targeting *S1PR3* or treated with TY-52156 (a potent S1PR3 antagonist). Protein and flow cytometry results revealed that S1PR3 suppression was similar to that of *SPHK1* silence, and induced G1/S phase arrest. As expected, S1P replenishment did not contribute to the G1/S phase arrest, as well as the reactivation of cyclin proteins and the downstream PBX1 expression, suggesting the possible bridge role of S1PR3 in SPHK1-mediated cell function (Figure 5D,E). Meanwhile, the similar tendency was also confirmed in another NSCLC cell line H226 (Figure S1C–E). Moreover, IHC staining showed that the higher expression level of S1PR3 was detected in NSCLC specimens compared with the adjacent control (Figure S1H), and the S1PR3 level was also proven to be positively correlated with tumor progression [32].

Above all, we proved that PBX1 participated in sphingolipid-regulated cell cycle progress, so PBX1, as a transcription factor, would in turn affect the transcription and expression of sphingolipid. Interestingly, protein analysis showed that cells with *PBX1* silence significantly decreased S1PR3 expression, while *PBX1* overexpression obviously up-regulated S1PR3 expression (Figure 5G and Figure S1G). The TIMER database (http://timer.cistrome.org/, accessed on 1 July 2022) further validated a positive correlation between the two proteins in lung cancer (Figure 5F and Figure S1F), which led us to wonder whether the transcription factor PBX1 promoted the S1PR3 expression. To this end, we acquired the promoter sequence of S1PR3 in the Ensembl online website (https://asia.ensembl.org/index.html, accessed on 5 May 2022) to detect whether PBX1 could directly bind and promote the transcription of S1PR3. A potential binding site for PBX1 (CGCTCAATCATG) was discovered in the promoter region of S1PR3 (Figure S3). The former and revised primers were designed around the potential binding motif, and the results of ChIP PCR illustrated that PBX1 did bind to the S1PR3 promoter region (Figure 5H). This may be the reason why S1PR3 was down-regulated in cells with *SPHK1* silence, perhaps through the down-regulation of PBX1 expression (Figure 5C). Taken together, these results supported the S1PR3-mediated SPHK1/PBX1 axis regulating cell cycle progression, and PBX1, in turn, regulating S1PR3 transcription, thus establishing a feedback loop between PBX1 and S1PR3.

Among the signaling pathways initiated by S1PRs, Akt and Erk signaling have been reported to play a role in regulating the cell cycle and cell survival [33,34]. We then examined whether the two signaling pathways participated in the SPHK1/PBX1-regulated cell cycle progress. Protein analysis showed that the decreased expression of PBX1 and the cyclin markers could be induced by the treatment of PF-543, TY-52156 and wortmannin (PI3K/Akt antagonist), while MEK1/2 inhibitor AZD6244 made no difference to the cyclin markers, suggesting that Akt signaling might be involved in the SPHK1/PBX1 axis-regulated cell cycle (Figure S2A,B). It was supported that the feedback loop between PBX1 and Akt was mutually beneficial for maintaining HF-MSCs in a highly proliferative state with multipotential capacity [26]. Meanwhile, cells with different levels of PBX1 expression appeared to be irrelevant to the p-Akt expression, suggesting PBX1 might be downstream of Akt signaling (Figure 5G and Figure S1G). Zhou et al. elucidated the regulatory mechanism upstream of PBX1 in dNK cells, in which EVT-derived HLA-G promoted the activation of Akt1 driving the expression of PBX1 [35], which was consistent with our study that PBX1 was located downstream of the Akt signaling pathway.

### 2.6. PF-543 Suppressed Tumor Growth and Induced Cell Cycle Arrest in H460 Xenograft Tumor

**Figure 6:**
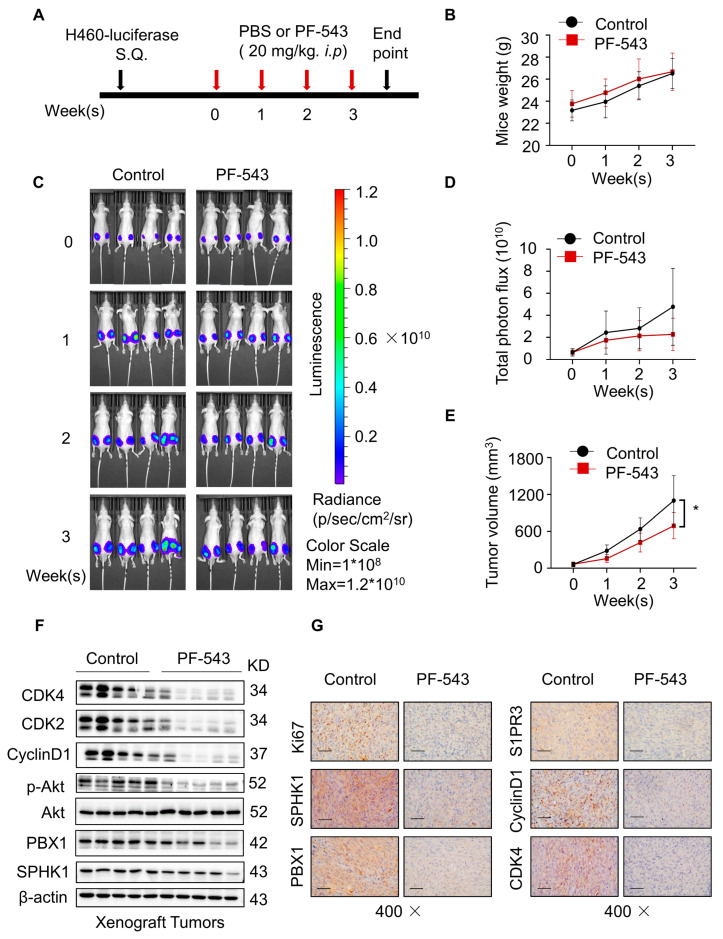
PF-543 suppressed tumor growth and induced cell cycle arrest in H460 xenograft tumor. (**A**) BALB/c male nude mice were inoculated with luciferase-expressing H460 cells (2.5 × 10^6^) subcutaneously (*n* = 4 in each group). Schematic modelling of the treatment to mice. (**B**) Mice were randomized into two groups and subjected to treatment PBS or PF-543 intraperitoneally (20 mg/kg body weight). Changes in body weight of mice during drug administration. (**C**) Bioluminescence intensity was monitored for each group during drug administration. (**D**) Total photon flux was quantified for each group during drug administration. (**E**) The volume of xenograft tumor in nude mice. The calculation formula of tumor volume is: V = (L × W^2^)/2. (*n* = 4 for each group). * *p* < 0.05, Control versus PF-543 at 3 week, Student *t*-test. (**F**) Western blot detected tissue protein expression of SPHK1, PBX1, p-Akt, Akt, CDK4, CDK2 and CyclinD1 in five randomly selected, paired tumor tissues from each group. (**G**) IHC staining with antibodies against Ki67, SPHK1, PBX1, SIPR3, CyclinD1 and CDK4 in control and PF-543 group, magnification 400×, bar = 50 µm.

To further assess the effects of SPHK1 in vivo, we subcutaneously injected equal amounts of luciferase-expressing H460 cells (2.5 × 10^6^) into BALB/c male nude mice. After 10 days, mice were randomized into two groups and subjected to treatment with PBS or PF-543 intraperitoneally (20 mg/kg body weight) every day for 3 weeks following the schedule (Figure 6A). Bioluminescence intensity was monitored and relative photon flux was quantified at the indicated times. Compared with the control group, we observed that PF-543 treatment displayed a lower bioluminescence intensity growth rate (Figure 6C,D) and diminished tumor volume (Figure 6E) but had no obvious effect on the body weight of nude mice at the specified concentration (Figure 6B). At the end point of the experiment, tumors were removed for further tissue proteins and IHC staining analysis. Protein results showed that PF-543 treatment significantly down-regulated the PBX1 expression and the phosphorylation of Akt, as well as the lower expression of the cyclin makers CDK4, CDK2 and CyclinD1 (Figure 6F). IHC staining further confirmed that the levels of Ki67, SPHK1, PBX1, S1PR3 and cyclin-related proteins were markedly reduced in tumors derived from the PF-543 treatment group (Figure 6G). Overall, these in vivo results corroborated the potential role of the SPHK1/PBX1 axis in the cell cycle regulation, which was consistent with in vitro studies.

## 3. Discussion

Sphingolipids, a unique group of bioactive lipids, have been demonstrated to play a role in the oncogenic process, which is associated with maintaining the equilibrium between the pro-survival and the apoptotic signaling of cells [36]. The propensity of S1P to oppose apoptosis pathways and to promote malignant phenotypes coupled with the exaggerated expression of SPHK1 in some human tumor specimens and a negative correlation between SPHK1 mRNA levels and survival have prompted a heated suggestion that SPHK1 serves not only as a predictive biomarker, but also as a potential target in tumor therapy. As a part of the sphingolipid metabolism, SPHK1 is the rate limiting enzyme of the rheostat and maintains the dynamic balance of the sphingolipid rheostat to reach relative homeostasis under normal physiological conditions [37], whereas elevated SPHK1 expression has been found in many types of cancer and leads to increased cell proliferation, migration capability, invasiveness, angiogenesis and inflammation with different oncogenic mechanisms [38]. It was reported that the activated SPHK1/Akt/NF-κB signaling pathway promoted cell proliferation, cell cycle G1/S transition and reduced the apoptosis and chemosensitivity of pancreatic cancer cells [39]. Moreover, an SPHK1-driven NF-κB/IL-6/STAT3/S1PR1 amplification loop, was also essential for the development and progression of colitis-associated cancer [40]. As for NSCLC, studies showed that miR-495-3p reprogramed the sphingolipid rheostat towards ceramide by targeting SPHK1 and induced lethal mitophagy to suppress NSCLC tumorigenesis [41]. The research here reported the accumulation of SPHK1 in NSCLC with the potential role in cell cycle regulation, functionally through the activation of the downstream effector PBX1, and PBX1 in turn directly promoted the expression of S1PR3, thus activating the sphingolipid metabolic circuit. Homeodomain transcription factor PBX1 orchestrates a complicated gene expression network and synergistically interacts in a temporal and spatial manner to maintain cells with self-renewal, repopulation and reprogramming [42]. In this study, we demonstrated that the SPHK1/PBX1 axis induced the activation of cyclin-dependent kinases, thereby promoting NSCLC tumorigenicity in vitro and in vivo, which was in agreement with a previous study showing that the overexpression of PBX1 in HF-MSCs promoted the progression of the cell cycle from G0/G1 to the S phase [43]. In addition to the regulatory role of the SPHK1/PBX1 axis in the cell cycle, SPHK1/S1P signaling is also known to be involved in hyperoxia-mediated ROS generation [44]. Huang et al. demonstrated a critical role for SPHK1/S1P signaling in TGF-β-induced Hippo/Yes-associated protein (YAP) 1 activation and mtROS generation, resulting in fibroblast activation and pulmonary fibrosis induction [45]. Meanwhile, PBX1 was also reported to attenuate HF-MSC senescence and apoptosis by alleviating ROS-mediated DNA damage [46], as well as the ROS production regulation in lung cancer cells [47]. However, in our research, whether there is a relationship between the SPHK1/PBX1 axis and the changes in intracellular oxidative stress needs to be further studied. In conclusion, these findings all support a potential oncogenic role of the SPHK1/S1P axis in lung diseases, and understanding its carcinogenic mechanism may bring new insights into the treatment of NSCLC or other diseases.

As mentioned above, the S1P-related pathway orchestrates diverse cell functions by acting in an “inside-out” manner through five S1P receptors, S1PR1-5, or as an intracellular secondary lipid messenger. In mammals, S1PR1, S1PR2 and S1PR3 are ubiquitously expressed in all tissues, while S1PR4 and S1PR5 are tissue-specific, with S1PR4 being highly expressed in lymphoid tissues and blood cells, and S1PR5 mostly presenting in the brain, skin and natural killer cells [48]. Deregulation of the S1P signaling pathway contributes to the development and progression of a variety of cancer types, and the altered expression of SPHK1 and the S1PRs are common mechanisms. S1PR1 overexpression significantly induced the expression and activity of urokinase plasminogen activator (uPA) and, thus, cell invasion in glioblastoma multiforme [49]. Similarly, the SPHK1/S1P/S1PR3 axis promoted the expansion of aldehyde dehydrogenase (ALDH)-positive cancer stem cells (CSCs) via ligand-independent Notch activation [50]. According to our work, NSCLC cells with *S1PR3* silence down-regulated PBX1 expression and induced G1 phase arrest, which failed to respond to the exogenous S1P stimulation, suggesting the bridge role of S1PR3 in the SPHK1/PBX1 axis-mediated cell function. Zhao et al. revealed that the levels of S1PR3 were significantly increased in human lung adenocarcinoma specimens, mechanistically, at least in part due to the TGF-β/SMAD3 signaling axis [51]. Our data supported that S1P catalyzed by SPHK1 activated PBX1 through S1PR3, and in turn PBX1 could bind to the S1PR3 promoter region and promote to the S1PR3 transcription, thus forming a positive feedback pathway among these two molecules. The evidence is compelling to support an oncogenic role for the SPHK1/S1P/S1PRs signaling cascade in carcinogenesis and efforts are now focused toward targeting this pathway in a therapeutic context. For example, SK1-I, a sphingosine analogue and competitive inhibitor of SPHK1, attenuated glioblastoma growth and proliferation in cell lines and xenograft models [52]. Pro-drug FTY720, a structural analogue of S1P but functional antagonist for S1PR1, targeted I2PP2A/SET and mediated lung tumor suppression via the activation of PP2A-RIPK1-dependent necroptosis [53]. Sphingomab, a novel anti-S1P monoclonal antibody (mAb), was used to inhibit systemic S1P signaling and attenuate lung metastasis [7]. Studies on SPHK1 inhibitors have made some progress, while no ideal SPHK1 inhibitor is currently used for treating cancer in the clinic, and there are also other compounds including S1PR1 and S1PR3 (VPC03090) or S1PR2 (AB1) antagonists under preclinical evaluation [54,55]. Although there are limited studies regarding the application of these inhibitors in NSCLC, there is no denying that targeting the SPHK1/S1P/S1PRs signaling cascade presents a potential treatment prospect in related diseases including NSCLC.

Despite the results obtained, there are still some questions worth our attention. Firstly, elevated SPHK1 expression has been observed in multiple types of cancer, but the molecular mechanism of SPHK1 up-regulation in tumors remains unclear. The activation of SPHK1 is mediated by three known ways, phosphorylation of the Ser225 site of SPHK1 by extracellular-regulated protein kinases, by external stimuli including growth factors and proinflammatory factors, and by the up-regulation of transcription levels, especially the epigenetic regulation [37]. Xia et al. demonstrated that miR-124 down-regulated SPHK1 expression by directly targeting its 3′-untranslated region (3′-UTR) and that miR-124 expression was inversely correlated with SPHK1 expression in gastric cancer samples [56]. Thus, the dysregulation of non-coding RNA caused by abnormal DNA methylation may be an important reason for the high expression of SPHK1 in tumors [6]. Secondly, our research revealed that SPHK1 exerted an effect on the cell cycle by regulating the PBX1 expression. However, the mechanism for how the SPHK1/PBX1 axis affects cyclin proteins and how it participates in the cell cycle progression remain unclear. Lin et al. reported that the transcription factor E2A-PBX1 suppressed the expression of the cell cycle inhibitor CDKN2C by mediating the epigenetic regulator SETDB2’s expression, thus establishing an oncogenic pathway in ALL [57]. Similarly, the cycle regulator CyclinD1 was maintained as a target gene of the JAK2/STAT3 signaling pathway subjected to the regulation of PBX1 in clear cell renal carcinoma [28]. However, in our study, whether PBX1 regulates the expression of cyclin proteins in these ways needs to be further explored. Thirdly, among the downstream signaling pathways initiated by S1P, the Akt signaling pathway seemed to participate in the SPHK1/S1PR3/PBX1 axis to regulate cell cycle progression. Meanwhile, the Erk pathway was also involved in the regulation of PBX1 expression, and whether the Erk pathway was involved in PBX1 mediated other malignant phenotypes and pathological processes of tumor cells. Studies have revealed that PBX3, the homologous family protein of PBX1, promoted the migration and invasion of glioblastoma and colorectal cancer via activation of the MAPK/ERK signaling pathway, which might bring new insights into the oncogenic role of PBX1 in NSCLC [58,59]. In addition, it is known that sphingolipid homeostasis is highly regulated by multiple metabolic enzymes and whether the inhibition of SPHK1 could cause compensatory changes in other metabolic enzymes in the sphingolipid pathway. The contribution of S1P in cells is regulated by its synthesis catalyzed by SPHKs and catabolism by S1P phosphatases or S1P lyase (S1PL); thus the combination treatment of S1P synthesis inhibition together with its catabolism promotion may bring potential benefits for tumor therapy [60].

In summary, this study linked the key enzyme of the sphingosine metabolism with the transcription factor PBX1 for the first time, and illustrated the potential regulatory role of the SPHK1/S1PR3/PBX1 axis in the cell cycle and a feedback interaction loop between PBX1 and S1PR3 in NSCLC. Our study gave a new enlightenment to the molecular mechanisms for sphingosine metabolism in the development of NSCLC and may provide new ideas for the treatment of NSCLC.

## 4. Materials and Methods

### 4.1. Reagents

The main reagents used in this experiment include PF-543 citrate (SPHK1 inhibitor; MedChemExpress, HY-15425A, Monmouth Junction, NJ, USA), PD98059 (MEK1/2 inhibitor; MedChemExpress, HY-12028, Monmouth Junction, NJ, USA), S1P (Cayman chemical, 9002921, Ann Arbor, MI, USA), TY-52156 (S1PR3 antagonist; Cayman chemical, 19119, Ann Arbor, MI, USA), wortmannin (PI3K/Akt antagonist; Cayman chemical, 10010591, Ann Arbor, MI, USA). The specific primary antibodies include against SPHK1 (1:1000, CST, 12071S, Danvers, MA, USA), PBX1 (1:1000, CST, 4342S, Danvers, MA, USA), Phospho-Akt (Ser 473; 1:1000, CST, 4060S, Danvers, MA, USA), Akt (1:1000, CST, 4685S, Danvers, MA, USA), Phospho-p44/42 MAPK (Thr202/Tyr204; 1:1000, CST, 4370S, Danvers, MA, USA), S1PR3 (1:1000, Abcam, ab126622, Cambridge, UK), S1PR1 (1:1000, Abcam, ab23386, Cambridge, UK), CDK4 (1:1000, Santa Cruz Biotechnology, sc-23896, Dallas, TX, USA), CDK2 (1:1000, Santa Cruz Biotechnology, sc-6248, Dallas, TX, USA), CDK1/CDK2 (1:1000, Santa Cruz Biotechnology, sc-53219, Dallas, TX, USA), CyclinD1 (1:1000, Santa Cruz Biotechnology, sc-8396, Dallas, TX, USA), β-actin (1:1000, Santa Cruz Biotechnology, sc-47778, Dallas, TX, USA), goat anti-rabbit and anti-mouse horseradish peroxidases (HRPs; 1:5000, Proteintech, B900610 and B900620, Chicago, IL, USA).

### 4.2. Cells

The human lung adenocarcinoma cell lines A549, H1299, H1792 and H1975, the human lung squamous cell carcinoma cell lines H226 and H520, the lung large cell carcinoma cell line H460 and the normal lung epithelial cell line BEAS-2B were purchased from ATCC. A549 and BEAS-2B cells were cultured in Dulbecco’s modified Eagle’s medium (DMEM) containing 10% FBS, 1% penicillin/streptomycin (Solarbio, P7630, Beijing, China). H1299, H1792, H1975, H226, H520, H460 cells were cultured in RPMI 1640 medium containing 10% FBS, 1% penicillin/streptomycin. All cultures were maintained at 37 °C with a 5% CO_2_ atmosphere in a humidified incubator.

### 4.3. shRNA/siRNA

The lentiviral SPHK1 shRNA plasmid or shRNA control plasmid (GenePharma, Shanghai, China) was transfected into HEK293T cells together with auxiliary plasmids pLP1, pLP2 and VSV-G to package lentivirus. Then indicated cell lines were established with stable SPHK1 knocked down or its scrambled controls. Single colonies were obtained after 2 weeks of 2.5 μg/mL puromycin selection. Cells were seeded at 50% confluence in six-well plates overnight and then transfected with either 50 nM siRNA including si*SPHK1*, si*PBX1*, si*S1PR3* (Tsingke, Beijing, China) or 2.4 μg PBX1-expressing plasmid (RuiBo, Guangzhou, China), and its negative control siRNA, or pcNC control plasmid using Lipofectamine 2000 Transfection Reagent (Invitrogen, 11668027, Waltham, MA, USA) according to the manufacturer’s instructions.

### 4.4. Immunoblotting Assay

The total protein was extracted using RIPA lysate, and the concentration was measured via bovine serum albumin (BSA) kit (Thermo Fisher Scientific, Waltham, MA, USA). Equal amounts of protein were mixed with 4×loading buffer and separated by 6–15% SDS-PAGE, then transferred onto polyvinylidene fluoride (PVDF) membranes (Millipore, HATF00010, Billerica, MA, USA), which were blocked with 3% Bovine Serum Albumin (BSA) for 60 min. The membrane was incubated with specific primary antibodies overnight at 4 °C. Then the blots were washed with TBS + 0.1% Tween-20 (TBST) 3 times (10 min per time) and incubated with peroxidase-conjugated secondary antibodies for 60 min at room temperature. The TANON image software (Beijing YuanPingHao Biotech, Beijing, China) was used to detect the enhanced chemiluminescence (Millipore, WBKlS0100, USA) of specific membranes.

### 4.5. Real-Time Quantitative PCR (qPCR)

Total mRNA was extracted using TRIzol reagent (CWBIO, CW0580S, Beijing, China) and RNA concentrations were determined using an ultraviolet spectrophotometer. cDNA was performed using a Prime Script™ RT Master Mix Kit (TransGen, AE301-02, Beijing, China) according to the manufacturer’s instructions. qPCR was performed using an Ultra SYBR Mixture Kit (CWBIO, CW0957C, Beijing, China) and a Bio-Rad CFX Maestro system. The reaction parameters were as follows: 95 °C for 15 min, 45 cycles of amplification with three steps: denaturation at 95 °C for 10 s, annealing at 55 °C for 20 s and extension at 72 °C for 30 s.

The primers used are listed below:

GAPDH forward 5′ AACGGATTTGGTCGTATTGG and

GAPDH reverse 5′ GGCTGCTGTCACCCATGAA

SPHK1 forward 5′ AACGGATTTGGTCGTATTGG and

SPHK1 reverse 5′ TCACTCTCTAGGTCCACATCAG

PBX1 forward 5′ CAGTGAGGAAGCCAAAGAGG and

PBX1 reverse 5′ CAGCTGTTTTGGCAGCATAA

S1PR1 forward 5′ GCCTACACAGCTAACCTGCTCTTG and

S1PR1 reverse 5′ TGGCGATGGCGAGGAGACTG

S1PR2 forward 5′ CCACCACCTCCTGCCACTCC and

S1PR2 reverse 5′ CACCGTGTTGCCCTCCAGAAAC

S1PR3 forward 5′ GATCCTCTACGCACGCATCTACTTC and

S1PR3 reverse 5′ ACACGCTCACCACAATCACCAC

S1PR4 forward 5′ GAAGCCGTAGACGCGGCTGG and

S1PR4 reverse 5′ GAAGCCGTAGACGCGGCTGG

S1PR5 forward 5′ GTGAGGTGGGAGCCATAGAA and

S1PR5 reverse 5′ TTGGCTGAGTCTCCCAGAGT

### 4.6. Cell Viability Assay

3-(4,5-Dimethylthiazol-2-yl)-2,5-diphenyltetrazolium bromide (MTT) reduction is one of the most frequently used methods for measuring cell proliferation and cell viability. Cells were seeded into 96-well plates at a density of 4 × 10^3^ cells per well in 100 μL of medium containing 10% FBS for 12 h, and fresh 1% medium containing PF-543 at different concentrations (2.5–50 μM) was incubated with cells for 48 h. The medium was removed, and the cells were cultured with MTT (0.5 mg/mL, 100 μL MTT/well) for 2 h. Then, the absorbance of DMSO-dissolved blue formazan crystals was read and quantitated. Values are expressed as the means ± SD; *n* = 5 /group. IC_50_ (inhibitory concentration causing a 50% response) of drug dose response curves for cell lines was calculated using the log (inhibitor) vs response method in GraphPad Prism (Version 8.3.0) according to the cell viability values obtained above.

### 4.7. Wound-Healing Assays

Cells were seeded in a 6-well plate at a density of 1 × 10^5^ cells per well. A 1000 μL micropipette tip was used to make a vertical scratch in the well center, then the cells were cultured in the absence or presence of PF-543 (15 μM) in 1% FBS culture medium. The scratch width in each well was observed and the pictures in different time periods obtained by light microscope. The data were analyzed with Image-J software.

### 4.8. Colony Formation Assay

Cells were seeded in a 6-well plate at a density of 2000 to 3000 cells per well and treated with 15 μM PF-543 for 72 h, followed by culturing in RPMI-1640 with 10% FBS for 10–14 days. Colonies were then fixed with 10% formalin for 5 min and stained with 0.05% crystal violet for 15 min. The data were analyzed with Image-J software.

### 4.9. Transwell Migration Assay

Cells were starved for 24 h, and 8 × 10^4^ cells were resuspended in 200 μL serum free media and then transferred into the upper chamber of a 24-well plate with 750 μL fresh medium containing 10% FBS in bottom chamber. After 48 h, the inserts were washed with PBS, fixed with 10% formalin for 5 min and stained with 0.05% crystal violet for 15 min. The data were analyzed with Image-J software.

### 4.10. Cell Cycle Analysis

Cells were seeded at 50% confluence in six-well plates overnight and then treated with fresh 10% medium containing PF-543 (15 μM), S1P (5 μM) or TY-52156 (5 μM) for 48 h. Cell cycle analysis was performed with cell cycle staining kit (Multi Sciences, CCS012, Hangzhou, China) according to the manufacturer’s instructions. Finally, flow cytometry analysis of cells was performed on a BD FACS Aria II flow cytometer (BD Biosciences, LSR Fortessa, Franklin, NJ, USA).

### 4.11. EdU Proliferation Assay

Cells were seeded at 50% confluence in six-well plates overnight and then transfected with 50 nM si*SPHK1* or S1P (5 μM) for 24 h. Cell proliferation was evaluated using the EdU Proliferation Kit (Thermo Fisher Scientific, Waltham, MA, USA) according to the manufacturer’s instructions. DAPI (Solarbio, S2110, Beijing, China, 10 μg/mL) was used to stain nucleus. Then, slides were washed and mounted with anti-fade mounting medium, and the fluorescence intensity was revealed by confocal fluorescence microscope (Leica, TCS SP8, Allendale, NJ, USA). The representative images by 630× magnification are presented.

### 4.12. Immunohistochemistry Staining

The tumor tissues were fixed with 4% paraformaldehyde before dehydration with gradient (70%, 80%, 95% and 100%) alcohol and xylene, and embedded in paraffin. Immunohistochemical staining was performed with the paraffin sections according to DAB working solution (Solarbio, DA1010, Beijing, China), and hematoxylin was used to stain cell nucleus. Primary antibodies anti-SPHK1, anti-PBX1, anti-S1PR3, anti-CDK4, anti-CyclinD1 and anti-Ki67 (CST, 9449, Danvers, MA, USA) were diluted at 1:200, and incubated on slides overnight at 4 °C, then imaged at ×200 magnification or at ×400 magnification.

### 4.13. Enzyme-Linked Immunosorbent Assay (ELISA)

Cell supernatant and cell precipitate were collected, and cell precipitate was suspended with PBS, ultrasound or repeated freeze–thaw lysed cell membrane to release cell contents. The contents of intracellular and extracellular S1P were determined using an S1P ELISA kit (BlueGene, Shanghai, China) according to manufacturer’s instructions. Values are expressed as the means ± SD; *n* = 3.

### 4.14. Chromatin Immunoprecipitation (ChIP)

ChIP assay was performed using the ChIP Chromatin Immunoprecipitation Kit (Absin, abs50034, Shanghai, China) with anti-PBX1 antibody according to the manufacturer’s instructions. Primers for the amplification containing PBX1-binding site-specific region of S1PR3 promoter (nucleotides −422 bp to −272 bp) were forward 5′ GAATGCACGGTCGGATAA and reversed 5′ CCATGATTGAGCGAACAC, with an expected size of 151 bp.

### 4.15. Xenograft Tumor Model

All experiments involving mice were approved by the Animal Care Committee of Nankai University, Tianjin, China (Animal Ethics Number, 2022-SYDWLL-0005000). BALB/c nude male mice at 6 weeks of age (22–25 g) were maintained under specific pathogen-free conditions (animal certificate number, SYXK [JIN]-2019-0001). Equal number (2.5 × 10^6^) of luciferase-expressing H460 cells were resuspended in 0.1 mL of PBS and then were injected subcutaneously into the left and right flanks of mice (*n* = 4 for each group). When the tumor volumes reached about 200 mm^3^ (volume = length × width^2^/2), the mice were randomly divided into two groups. The administration group was treated with PF-543 (20 mg/kg, i.g.), diluted in PBS. The control group was treated with an equal volume of PBS, and the medicine was given every day. Primary tumors were measured using Vernier calipers. Mice were given the substrate D-luciferin (PerkinElmer, 122799, Waltham, MA, USA) by intraperitoneal injection (150 mg/kg), and tumors were assessed using bioluminescence imaging (BLI; PerkinElmer, IVIS Spectrum, USA) every week. Animals were head-fixed and anaesthetized with isoflurane (∼1%) during imaging. After three weeks, tumors were harvested for the experiments.

### 4.16. NSCLC Patient Lung Tissue

NSCLC patient lung tissues were obtained from the Tianjin Cancer Hospital, China. Written informed consent was obtained from each patient in this study, and protocols were approved by the ethical committee of Tianjin Cancer Hospital (Medical Ethics Number, NKUIRB2022106).

### 4.17. Statistical Analysis

Results are shown as means ± SD of at least three independent experiments and were analyzed using GraphPad Prism (Version 8.3.0). Student’s *t*-test was used to compare significance of the difference between two groups, and two-way ANOVA was used for multiple-group comparisons. * *p* < 0.05, ** *p* < 0.01, *** *p* < 0.001, **** *p* < 0.0001.

## Data Availability

All data generated or analyzed during this study are included in this article and are available from the corresponding author upon reasonable request.

## Supplementary Materials

The supporting information can be downloaded at: https://www.mdpi.com/article/10.3390/ijms232112741/s1.
