# Supplementary material for: Targeting SPHK1/PBX1 Axis Induced Cell Cycle Arrest in Non-Small Cell Lung Cancer
Source: Int J Mol Sci. 2022 Oct 22;23(21):12741. doi: 10.3390/ijms232112741 (PMC9657307; doi:10.3390/ijms232112741)
Supplement: Supplementary file 1 [file ijms-23-12741-s001.zip › ijms-1933602-supplementary.pdf]

Targeting SPHK1/PBX1 Axis Induced Cell Cycle Arrest in Non-Small Cell Lung Cancer

Zhoujun Lin [1], Yin Li [1], Xiao Han [1], Zhenkun Fu [1,2], Zhenhuan Tian [3,*] and Chenggang Li [1,*]

1 State Key Laboratory of Medicinal Chemical Biology and College of Pharmacy, Nankai University, No. 38 Tongyan Road, Jinnan District, Tianjin 300350, China
2 Heilongjiang Provincial Key Laboratory for Infection and Immunity, Department of Immunology, Wu Lien-Teh Institute, Heilongjiang Academy of Medical Science, Harbin Medical University, Harbin 150081, China
3 Department of Thoracic Surgery, Peking Union Medical College Hospital, No. 1 Shuaifuyuan, Dongcheng District, Beijing 100730, China
* Correspondence: tianzhenhuan@pumch.cn (Z.T.); lichenggang@nankai.edu.cn (C.L.)

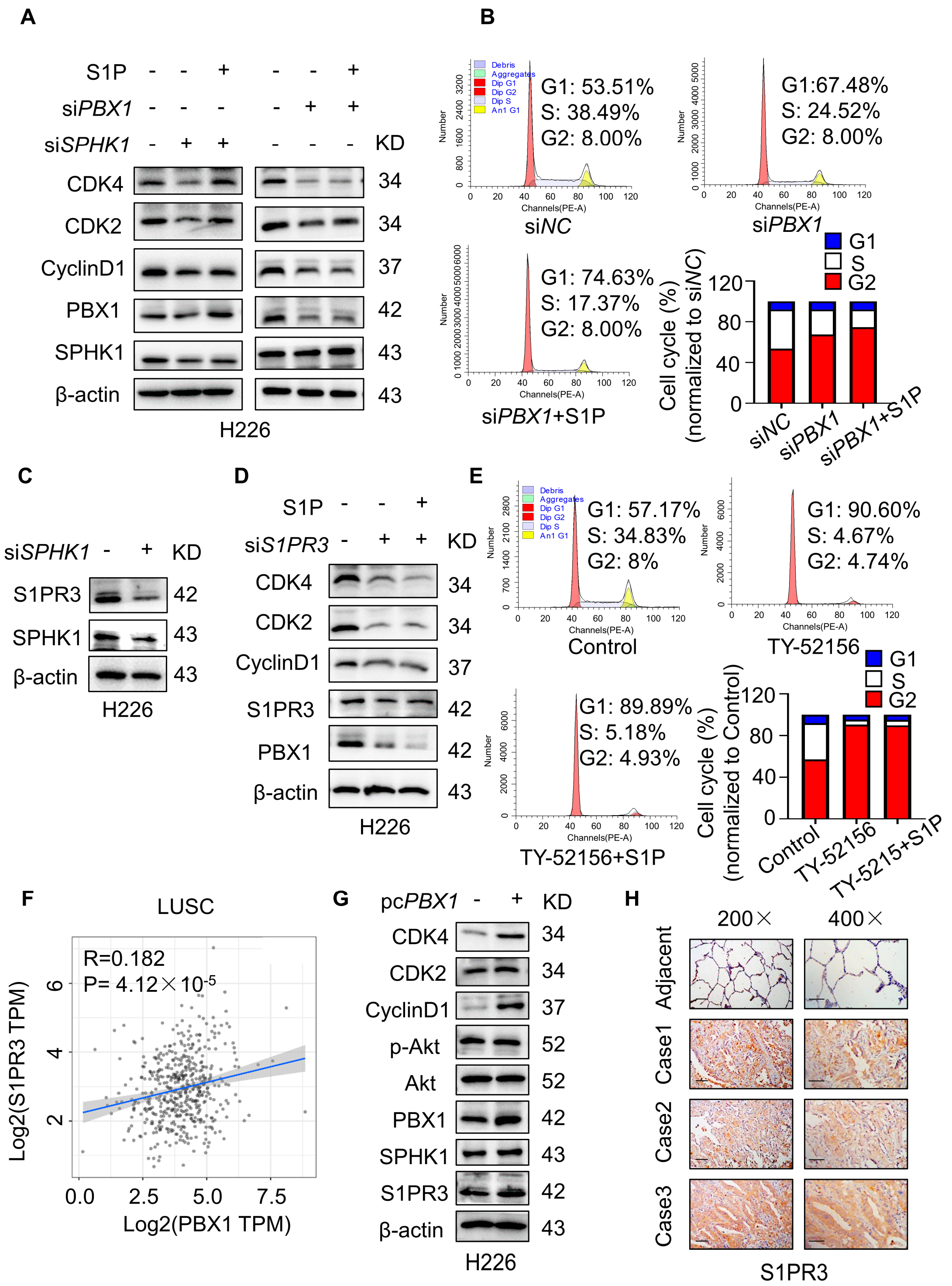

Figure S1. SPHK1/S1PR3/PBX1 aixs regulated cell cycle progress in H226 cells.

(A) Western blot detected the change of SPHK1, PBX1, CDK4, CDK2 and CyclinD1 in H226 cells with *SPHK1* or *PBX1* silence (siRNA 50 nM) in the presence or the absence of S1P (5 μM). **(B)** Flow cytometry analyzed cell cycle distribution in H226 cells with *PBX1* silenced (siRNA 50 nM) in the presence or the absence of S1P (5 μM). **(C)** Western blot detected the expression of S1PR3 and SPHK1 with *SPHK1* silence (siRNA 50 nM) in H226 cells. **(D)** Western blot detected the the expression of S1PR3, SPHK1, PBX1, CDK4, CDK2 and CyclinD1 in H226 cells with *S1PR3* silence (siRNA 50 nM) in the presence or the absence of S1P (5 μM). **(E)** Flow cytometry analyzed cell cycle distribution in H226 cells with TY-52156 (5 μM)

treatment in the presence or the absence of S1P (5 μM). **(F)** Gene correlation analysis between *S1PR3* and *PBX1* of LUSC in TIMER database. **(G)** Indicated proteins were detected in H226 cells with *PBX1* overexpression. **(H)** IHC analysis of S1PR3 protein in NSCLC tumors compared with adjacent normal control, magnification 200×, bar = 100 μm, 400×, bar = 50 μm.

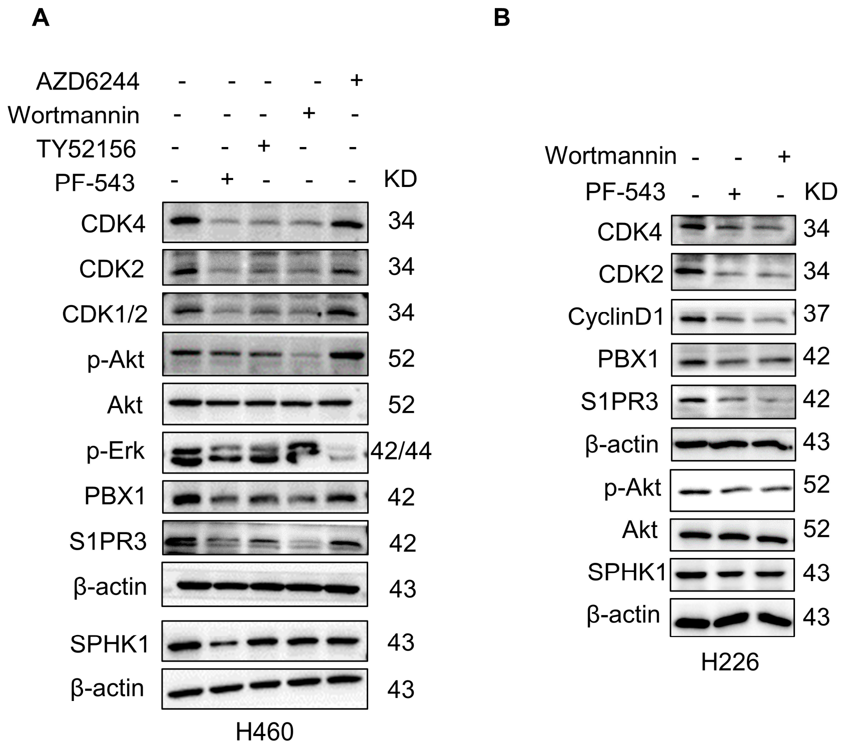

Figure S2. Akt signaling may participated in SPHK1 regulated cell cycle.

(A) H460 cells were treated with PF-543 (15 μM), TY52-156 (5 μM), Wortmannin (2 μM), or AZD6244 (0.2 μM) for 24 h and the protein expression of SPHK1, PBX1, S1PR3, p-Akt, Akt, p-Erk, CDK4, CDK2 and CDK1/2 were detected by western blot. **(B)** H226 cells were treated with PF-543 (15 μM) or Wortmannin (2 μM) for 24 h and and the protein expression of SPHK1, PBX1, S1PR3, p-Akt, Akt, CDK4, CDK2 and CyclinD1 were detected by western blot.

S1PR3 promoter region

-500 bp

AGACAGAAAGTAACGAGCGGCCTTTGCACAAACTCAACTCTCTGCAGCG
GCCCCTGCGGCCCAATCCTGCCGGCCCAGGAATGCACGGTCGGATAAC
AGGCCTCTCCTTGCTTTTTCGTCTTCTTCCTCCCGCTTCTCTCCTCTCTCC
TTTTCCTCCTCCTCCTTTTTCTTTAATTTTCTCCTTTCTCCCCAAGCTTCTA
CAGTTGTTTCTGTGTT**CGCTCAATCATG**GCCTTTGGCATTGATTGAAAATT
TGGCAGTTCCTTCTCTCGGAATTCTGTTTGAAGCCTTCATATGGATTTATT
ATTATTATTATTATTTTTGCCACACAAAAGGGGCAAATCCGCGTGAGGAA
GGGGGTCGCATGGGGGTCGGCTGAGATCTGCTGGCTGCCGGTCTAGGA
GGGGCGCTGCTCTGTGCCGGA

-80 bp

PBX1 predicted bingding motifs, **CGCTCAATCATG**

Figure S3. ChIP PCR of S1PR3 region designed for this study.

S1PR3 promoter region containing PBX1 predicted bingding motifs, CGCTCAATCATG.
